# Genotype-by-environment interactions affecting heterosis in maize

**DOI:** 10.1371/journal.pone.0191321

**Published:** 2018-01-17

**Authors:** Zhi Li, Lisa Coffey, Jacob Garfin, Nathan D. Miller, Michael R. White, Edgar P. Spalding, Natalia de Leon, Shawn M. Kaeppler, Patrick S. Schnable, Nathan M. Springer, Candice N. Hirsch

**Affiliations:** 1 Department of Agronomy and Plant Genetics, University of Minnesota, Saint Paul, Minnesota, United States of America; 2 Department of Agronomy, Iowa State University, Ames, Iowa, United States of America; 3 Department of Botany, University of Wisconsin, Madison, Wisconsin, United States of America; 4 Department of Agronomy, University of Wisconsin, Madison, Wisconsin, United States of America; 5 Department of Plant and Microbial Biology, University of Minnesota, Saint Paul, Minnesota, United States of America; University of Guelph, CANADA

## Abstract

The environment can influence heterosis, the phenomena in which the offspring of two inbred parents exhibits phenotypic performance beyond the inbred parents for specific traits. In this study we measured 25 traits in a set of 47 maize hybrids and their inbred parents grown in 16 different environments with varying levels of average productivity. By quantifying 25 vegetative and reproductive traits across the life cycle we were able to analyze interactions between the environment and multiple distinct instances of heterosis. The magnitude and rank among hybrids for better-parent heterosis (BPH) varied for the different traits and environments. Across the traits, a higher within plot variance was observed for inbred lines compared to hybrids. However, for most traits, variance across environments was not significantly different for inbred lines compared to hybrids. Further, for many traits the correlations of BPH to hybrid performance and BPH to better parent performance were of comparable magnitude. These results indicate that inbred lines and hybrids show similar trends in environmental response and both are contributing to observed genotype-by-environment interactions for heterosis. This study highlights the degree of heterosis is not an inherent trait of a specific hybrid, but varies depending on the trait measured and the environment where that trait is measured. Studies that attempt to correlate molecular processes with heterosis are hindered by the fact that heterosis is not a consistent attribute of a specific hybrid.

## Introduction

Heterosis, or hybrid vigor, refers to the phenomena in which the offspring of two inbred parents exhibits phenotypic performance beyond the mid-parent or best parent used to generate the hybrid. Heterosis has been observed in many plant and animal species [1, 2]. Notably, the heterosis of mules (the ability to perform more work with fewer resources) was widely utilized in agriculture prior to mechanization [3]. Inbreeding depression and heterosis in maize was initially documented by George H. Shull and Edward M. East [4–6]. The adoption of hybrid maize over open-pollinated varieties occurred remarkably fast due to improved yields, greater uniformity for machine harvesting, and increased durability under extreme abiotic stress. In just a four-year period of time, hybrid maize acreage went from less than 10% to over 90% in Iowa [7]. The widespread utilization of heterosis now shapes breeding programs for several agriculturally important species including maize and rice.

There is widespread interest in developing methods to characterize the molecular basis of heterosis, and to predict hybrid performance to increase the efficiency of hybrid breeding programs. Researchers have attempted to utilize genomic sequence [8], RNA expression levels of genes [2, 9, 10], sRNAs [11, 12], proteomic [13], and metabolomic [8, 14] data to predict or dissect heterosis [15]. While relationships have been identified using each of these data types, no data type is able to completely predict hybrid performance individually [16]. Attempts to predict hybrid performance are complicated by the fact that heterosis levels vary for different traits within the same hybrid [17].

Although plant breeders have noticed that hybrid genotypes are more stress tolerant than their inbred parents, there are few published reports to support this conclusion, particularly in environments with moderate rather than extreme levels of abiotic stress. In *Arabidopsis*, stress response gene expression networks have been shown to contribute to heterosis and the prediction of hybrid performance [18, 19]. While variation in levels of heterosis has been observed under different growing conditions, there are few studies that document changes in heterosis across diverse environmental conditions and traits.

In this study we measured 25 traits in 47 maize hybrids and their inbred parents grown in 16 different environments. The objectives of this study were to evaluate the extent of genotype-by-environment interaction for heterosis across a large number of traits that span developmental space and time, determine if there is a relationship between heterosis for the same trait at different developmental stages, and determine the contribution of inbred and hybrid variation to genotype-by-environment interactions for heterosis.

## Materials and methods

### Germplasm

Eleven inbred lines were selected representing important founders in commercial maize breeding programs including DK3IIH6 (PI 564754), LH145 (PI 600959), LH185 (PI 576171), LH198 (PI 557563), LH82 (PI 601170), PHB47 (PI 601009), PHK56 (PI 543842), PHK76 (PI 601496), PHN46 (PI 543844), PHP02 (PI 601570), and a recent release W606S. These inbred lines represent multiple heterotic groups including Iodent (DK3IIH6, PHP02), Non-Stiff Stalk (LH185, LH82, PHK76, PHN46, PHK56, W606S), and Stiff Stalk (LH145, LH198, PHB47). These lines, with the exception of W606S, are all commercial inbred lines that have expired Plant Variety Protection certificates, and thus represent elite maize germplasm. These inbred lines were crossed in a partial diallel to generate 47 hybrid genotypes (S1 Table).

To evaluate genetic diversity between the parental lines used to generate the hybrid genotypes, genetic similarity between the parents was calculated using whole genome identity by state [20] using 430,000 SNPs derived from RNA-sequencing [21].

### Field evaluations

Trials containing single row plots (3.35 m long and 0.76 m apart) were planted in a total of 16 environments in Iowa, Minnesota, and Wisconsin in the summer of 2015. The 16 environments were defined by location (5 separate locations), and management practices within location (planting date; high (70,000 plants ha^-1^) and low (20,000 plants ha^-1^) plant density). Arlington, WI and Waseca, MN had high and low planting densities, representing a total of four environments. Curtiss, IA, Kelly, IA, and St. Paul, MN had a factorial of high and low planting density and early and late planting at each site, representing a total of 12 environments (S2 Table). Within each location/management environment there were two replications and hybrids were blocked separately from inbred lines within each replication.

Twelve vegetative traits were measured on six representative plants per plot. These traits included plant height at 14, 21, 28, 35, 42, 49, 56, and 63 days after planting (not all time points were measured in all environments, see S2 Table) measured as the distance from the soil surface to the uppermost leaf tip when the leaves were pulled upright. Plant height at maturity was measured from the soil surface to the collar of the flag leaf, ear height at maturity was measured from the soil surface to the node subtending the uppermost ear. Leaf number above the ear, and leaf number below the ear (including senesced leaves) were counted after anthesis. Juvenile leaves were marked to allow leaf number including senesced leaves to be counted using previously described methods [21]. Days to anthesis (DTA) and days to silk (DTS) were measured on a per-plot basis as the day on which approximately half of the plants in the plot were shedding pollen and the day on which half of the plants in the plot had exposed silks, respectively. Custom computer algorithms were executed on the Open Science Grid computational resources [22] in a workflow managed by HTCondor software [23] to quantify eleven ear and kernel traits from digital images as previously described [24]. Six representative ears per plot were measured. Ear weight and grain weight was an average of the weight of the uppermost ear on the six representative plants in the plot and cob weight was measured on individual uppermost ears from the six representative plants in the plot (Table 1 and S3 Table). For all traits for which single plant measurements were taken, the same six representative plants were used for all measurements. See S2 Table for details on which traits were measured in each environment and S3 Table for raw phenotypic values.

**Table 1:** Summary of better parent heterosis (BPH) for 25 traits measured across 16 environments.

Trait	Abbreviation	Locations	Plots	Average %BPH	% Entries with BPH^a^
Plant Height 14 DAP	PHt14	4	169	20.2	95.3
Plant Height 21 DAP	PHt21	8	340	24.3	95.6
Plant Height 28 DAP	PHt28	12	528	27.9	98.1
Plant Height 35 DAP	PHt35	12	538	30.4	99.4
Plant Height 42 DAP	PHt42	12	538	33.8	99.8
Plant Height 49 DAP	PHt49	10	461	35.8	99.8
Plant Height 56 DAP	PHt56	8	282	38.5	100.0
Plant Height 63 DAP	PHt63	2	94	41.4	100.0
Plant Height Maturity	PHt	16	716	27.9	100.0
Ear Height Maturity	EH	16	716	36.5	99.3
Days to Anthesis^b^	DTA	16	670	-4.4	82.1
Days to Silk^b^	DTS	16	670	-5.0	87.0
Leaf Number Above Ear	LNA	12	538	7.6	53.9
Leaf Number Below Ear	LNB	8	375	3.1	20.0
Cob Width	CW	16	714	8.8	82.1
Cob Length	CL	16	714	20.3	93.1
Cob Weight	CWT	16	714	50.5	91.2
Ear Width	EW	16	710	13.5	95.4
Ear length	EL	16	713	18.6	91.6
Ear Weight	EWT	16	714	84.9	98.9
Kernel Row Number	KRN	16	713	7.2	58.6
Kernel Height	KH	16	710	26.1	89.6
Kernel Width	KW	16	709	8.7	59.4
Kernel Depth	KD	16	714	2.8	1.5
Per Plant Grain Weight	GWT	16	714	91.4	99.4

^a^Entry is the inbred/hybrid combination within an environment

^b^For these traits the better-parent performance was the lower value.

### Statistical analyses

Better-parent heterosis and percent better-parent heterosis (%BPH) were calculated for each trait and hybrid within each replicate block as

BPH=hybridphenotype-betterparentphenotype (1)

$$\%BPH=\frac{hybrid\;phenotype\;-\;better-parent\;phenotype}{better-parent\;phenotype\;}\times 100$$ (2)

BPH and %BPH were averaged across replicates within an environment. The average %BPH of the two replicates in each environment was used for subsequent analyses. For all traits except flowering time the higher parent was considered the better parent. For flowering time, the earlier parent was considered the better parent.

Phenotypic data were analyzed to partition variation using the model in Eq 3.

$$y_{ijk}=\mu +f_{k}+e_{i}+{r(e)}_{j(i)}+({ge)}_{k\times i}+\varepsilon_{ijk}$$ (3)

In Eq 3 *y*_*ijk*_ is the phenotype value of the *k*th genotype in *i*th environment of the *j*th replication; *μ* is the phenotypic mean across environments; *f*_*k*_ is *k*th genotype effect; *e*_*i*_ is the *i*th environmental effect; *r*(*e*)_*j*(*i*)_ is the *j*th replication effect nested within the *i*th environment; (*ge*)_*k*×*i*_ is the interaction effect of the *k*th genotype by the *i*th environment, and *ε*_*ijk*_ is the residual effect. All variables except μ were considered as random effects [25–28]. This analysis was done for inbred traits per se, hybrid traits per se, and heterosis for all 25 traits.

Broad-sense heritability (*H*^*2*^) was calculated as in Eq (4).

$$H^{2}=\frac{V_{G}}{V_{G}+V_{GxE}+V_{\varepsilon }}$$ (4)

*V*_*G*_ is the genetic variance, *V*_*G×E*_ is the genotype by environment variance, and *V*_*ε*_ is the error variance.

Pearson correlation coefficients and corresponding significance tests were conducted using PROC CORR in SAS 9.0 [29]. Correlation coefficients between traits and %BPH of traits were calculated on an environment basis using the average of the two replicates. The coefficient of variation (CV) for traits was calculated as the standard deviation divided by the unit (i.e. plot) mean. This is the most widely used parameter to quantify variability of traits with different units of measurement among individual plants and across environments [30]. The CV within plot was calculated from the phenotypic variation of the six plants in each plot divided by the plot mean. The CV across environments was calculated from the phenotypic variation across the environments divided by the mean across environments.

### Network visualization and clustering analyses

Cytoscape software [31] was used to visualize the correlation network among different traits. Pearson correlation coefficients of BPH between traits were used as the input value. Only correlation coefficients less than -0.3 or greater than 0.3 were shown. The *R* package “heatmap.2” was used to make the heatmap and to cluster the corresponding columns and rows. By default, “heatmap.2” uses a euclidean measure to obtain a distance matrix and complete agglomeration for clustering (https://www.rdocumentation.org/packages/gplots/versions/3.0.1/topics/heatmap.2).

## Results and discussion

### Better parent heterosis is variable across traits, environments, and developmental time

The majority of 25 measured traits exhibited significant genotype, environmental, and genotype-by-environment interaction effects in both the inbred lines and the hybrids across the 16 environments as well as for BPH (S4 Table). Better-parent heterosis was detected for all 25 traits to various degrees, and 16 of them exhibited BPH in more than 90% of hybrids (Table 1). Only two traits, leaf number below the ear and kernel depth, exhibited BPH in fewer than 50% of the hybrids. The average BPH varied substantially among the different traits (Table 1). Some traits such as grain yield per plant (GWT) exhibited BPH values greater than 90% BPH while other traits such as flowering time (DTA/DTS) exhibited a lower magnitude of BPH (-4.4 to -5.0%). However, for both of these traits the majority of hybrids exhibited BPH across all environments (Table 1).

**Fig 1:**
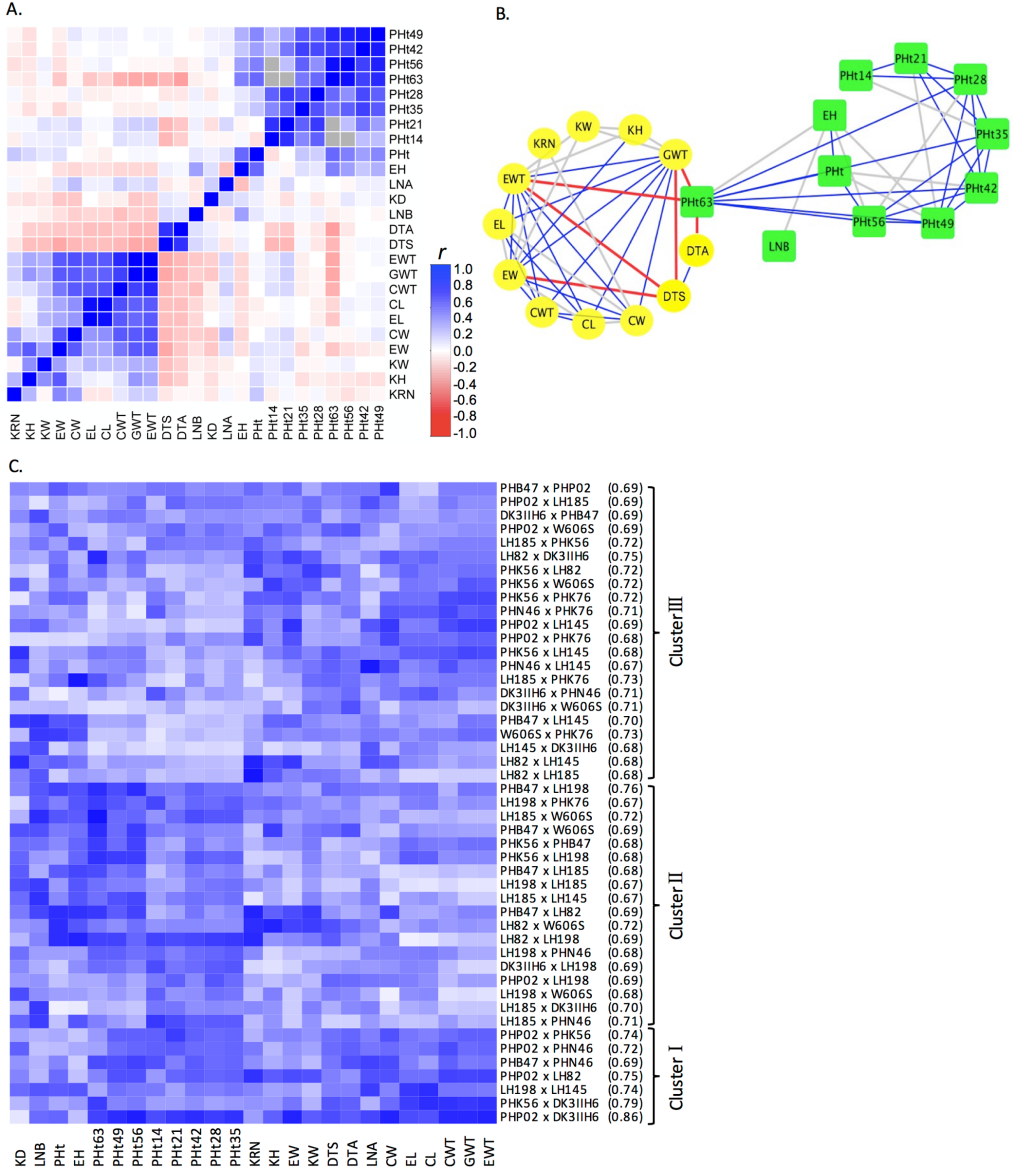
Better parent heterosis (BPH) comparisons for 25 traits and 47 hybrids across 16 environments. A) Pearson correlation coefficients (*r*) of BPH between traits; gray shaded cells indicate missing data. B) Network visualization of Pearson correlation coefficients of BPH between traits. Only correlation coefficients less than -0.3 or greater than 0.3 are shown. Traits in yellow circles and green rectangles are reproductive and vegetative traits, respectively. Red line, *r*<-0.3; gray line, 0.3<*r*<0.5; blue line, *r*>0.5. C) Average BPH rank scaled with white (highest BPH rank) to dark blue (lowest BPH rank). Hybrid genotypes are followed by the parental identity by state value.

The correlation of BPH across the traits studied varied substantially ranging from (*r* = -0.33 for ear weight (EWT) and plant height 63 days after planting (PHt63) to 0.99 for EWT BPH and GWT BPH; Fig 1A and 1B), similar to previous observations [17]. A network visualization of the correlations between BPH for distinct traits revealed several trends (Fig 1B). Strong positive correlations were observed within groups of traits that likely share a common genetic, physiological and developmental basis, including yield related traits (cob, ear, and kernel traits) and vegetative traits including plant height at 14 days after planting through maturity and ear height (Fig 1A and 1B). Days to silk and PHt63 BPH had strong negative correlations with GWT BPH, EWT BPH and ear width BPH because the better parent was the one that flowered early, and the hybrids flowered generally earlier than the better parent. PHt63 BPH was correlated with both vegetative and reproductive plant traits, connecting the two subgroups (Fig 1B).

The 47 hybrids could be assigned into three general clusters based on their relative heterosis performance (rank number) across the 25 traits (Fig 1C). The first cluster (n = 7) exhibited consistently lower BPH for all the traits relative to other hybrids and was enriched for within heterotic group hybrids (i.e. NSS x NSS; S5 Table). Hybrids in the second cluster (n = 18) showed relatively high heterosis for yield-related and flowering time traits, but lower heterosis for most of the vegetative traits, while hybrids in the third cluster (n = 22) were the opposite (Fig 1C).

Among the 47 hybrids genotypes, identity by state values ranged from 0.67 to 0.86 for the widest and narrowest crosses. Genotype clusters 1, 2, and 3 had identity by state (IBS) averages of 0.755, 0.694, and 0.702 respectively. The hybrid genotypes in cluster 1, which had the lowest relative heterosis across all traits, was composed of relatively narrow crosses. The hybrid genotypes in cluster 2, which had the highest amount of heterosis across yield-related and flowering traits, was composed of relatively wide SSS x NSS crosses. This supports the historical convention of breeders crossing between heterotic pools of unrelated inbreds to maximize heterosis for yield related traits. By nature of the partial diallel design that was used in this experiment, the hybrids that were generated were not independent, which is in part reflected in these IBD values. However, we do not believe that the results would be substantially different if each hybrid were generated from unique inbred lines due to the high level of IBS and identity-by-descent that is present in adapted elite inbred lines [32]. If we were to expand to, for example, tropical material where we might be able to achieve complete independence, issues related to local adaption and day length would be likely to confound any conclusions with regards to heterosis.

### There is low predictive capacity of heterosis over developmental time

Researchers have long been interested in the manifestation of heterosis at early stages of development [33–39]. Studies have shown heterosis of biomass production in Arabidopsis was established during early development [34, 36]. It is desirable to identify traits early in development that predict heterosis and yield at the end of the season. Previous reports indicate that traits measured at maturity showed the highest level of heterosis [40]. While it has been shown that heterosis could already be detected during early stages of maize seedling growth [41] and embryo development [33, 35], it has not been shown whether this early establishment of heterosis remains constant throughout time or changes with different developmental stages.

**Fig 2:**
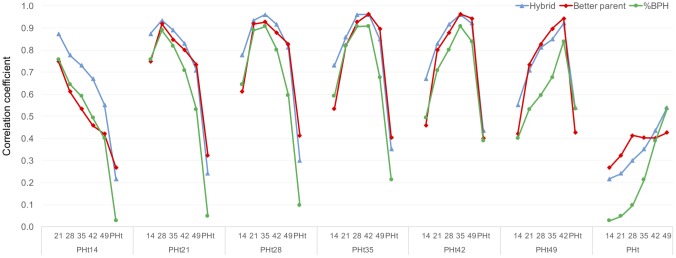
Correlation coefficient for percent better parent heterosis (%BPH), hybrid performance, and better-parent performance of plant height at different development stages in different environments. The numbers of 14–49 in x-axis indicate days after planting and PHt is plant height at physiological maturity.

To determine the potential of heterosis based on early developmental stages to predict heterosis at later development stages we measured heterosis for plant height at seven developmental stages ranging from 14 days after planting to anthesis. Plant height is one of the few morphological phenotypes that can be evaluated throughout developmental time. A low correlation was observed between heterosis for plant height at early developmental stages and at anthesis (Fig 2). However, final plant height was more highly correlated with measures at developmental stages closer to anthesis. Overall, our results indicate that heterosis measured at the seedling stage is not predictive of heterosis at the adult stage.

These low levels of correlation could potentially be a product of low correlation for the hybrid performance, the better parent performance, or both. To evaluate what drives this reduced correlation in heterosis over increased windows of developmental time, correlation coefficients for hybrid performance over time and inbred performance over time were overlaid with heterosis correlations (Fig 2). Both hybrid performance and inbred performance showed a similar tendency over time, indicating that both hybrid and better parent performance have a comparable effect on the lack of correlation from early stages of development to maturity.

We would not necessarily expect plant height at early developmental stages to correlate with plant height at later developmental stages. At early developmental stages plant height will be a function of leaf length and the rate of leaf production, while plant height later in development will be a function of internode length and leaf number. This difference in physiological basis for observed plant height may underlie the lack of correlation that is observed over developmental time. There may be other traits that show stronger correlation with early traits that are based on consistent physiological parameters throughout development. In the context of plant height, given the inability to predict heterosis levels, or even heterosis ranks, for the same trait collected at different stages of development it is likely to be quite difficult to predict adult plant traits from seedling traits or to relate specific heterosis mechanisms observed in the seedling to those contributing to variation in heterosis for this trait at maturity.

### Performance of hybrids is more stable than inbred lines within environments but not consistently among environments

Differential responses of maize hybrids and/or inbred lines to environmental stimuli will result in altered levels of heterosis across environments [30, 42]. Evidence from multiple species indicates that hybrids performance is more stable across environments than inbred performance [43]. This observation is consistent with the concept of “buffering” in which heterogeneous populations or heterozygous individuals are more stable than homogeneous populations or homozygous individuals [43–45]. To address this question across traits that span developmental space and time, we compared the stability of inbred and hybrid traits both within an environment and among environments.

**Fig 3:**
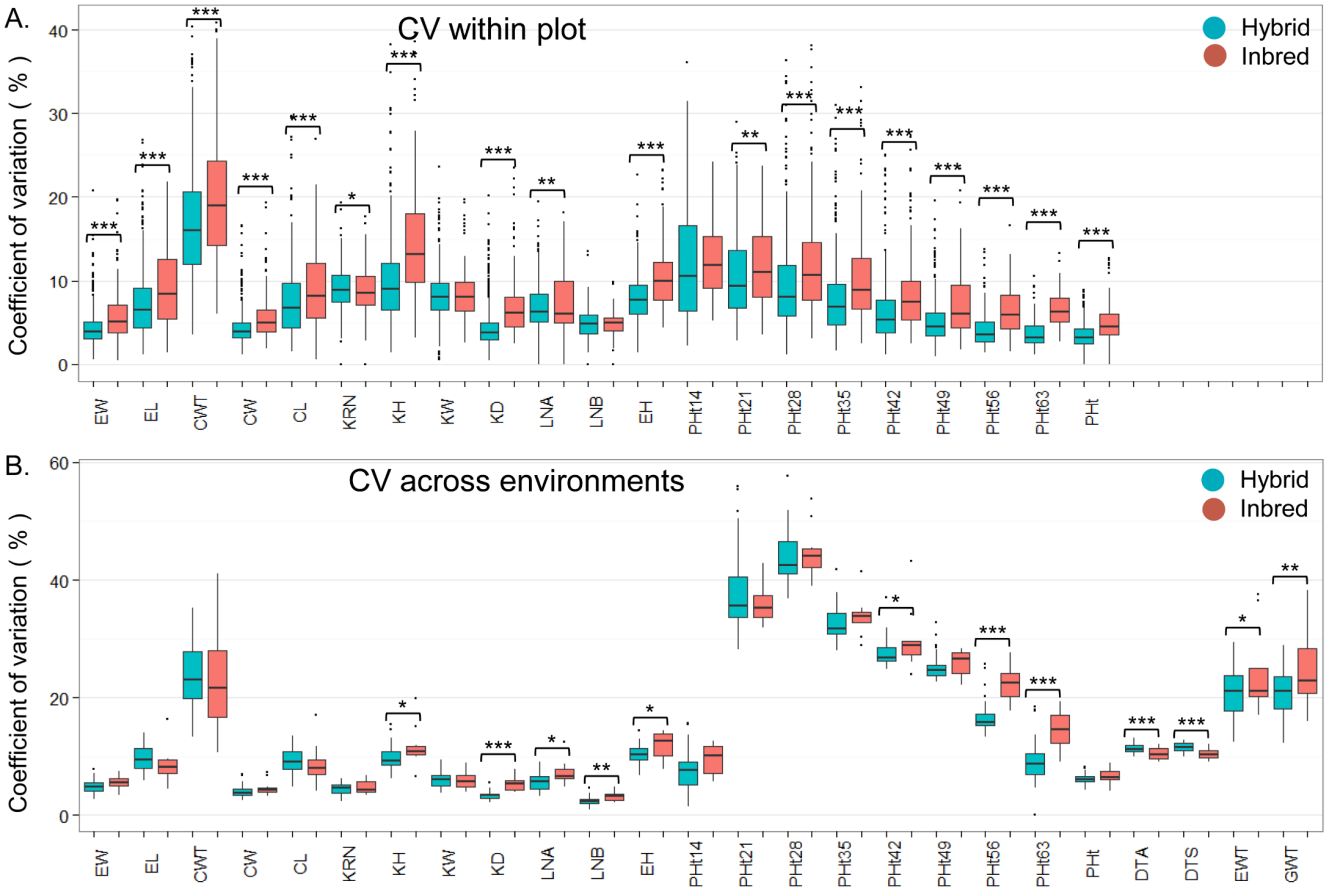
Coefficient of variation within and across environments for hybrid and inbred genotypes. A) Coefficient of variation within plot (6 plants were phenotyped within each plot). B) Coefficient of variation across all available environments for each trait. In each figure blue and red colors indicate hybrid and inbred, respectively. * significant at *p* = 0.05; ** significant at *p* = 0.01; *** significant at *p* = 0.001 in a two-tail t-test between the inbred and hybrid genotypes.

To evaluate stability across traits the CV was used. The within plot CV for inbred lines in this study was greater than the within plot CV for hybrids for nearly every trait measured (Fig 3A), providing evidence for greater variability of inbred lines within environments for most traits. We also assessed the CV among environments for each trait in the inbred and hybrid lines (Fig 3B). For ten of the traits the inbred lines exhibited a significantly higher CV than the hybrids, indicating that for these traits the instability of heterosis across environments was driven more by the instability of inbred lines. However, for flowering time traits (DTA and DTS), hybrids had significantly higher CV than inbred lines across the environments. The remaining 13 traits did not exhibit significant differences between the hybrid and inbred lines for the CV among environments. For the plant height measurements over developmental time, the CV among environments decreased throughout time for both inbred lines and hybrids, indicating increasing stability of both hybrids and inbred lines at later developmental stages. These results support the conclusion that performance of hybrids is more stable than inbred lines within environments but is not consistently more stable among environments across traits in contrast to what would be expected based on the buffering theory described above [43–45].

### Factors influencing environmental variation for heterosis are variable across traits

We were interested in assessing the factors contributing to the significant genotype-by-environment interaction effect on heterosis. For this question, we focused on GWT and plant height at maturity (PHt). These traits have variable heritability (S6 Table), and BPH for these traits were not significantly correlated across genotypes or environments (Fig 1).

**Fig 4:**
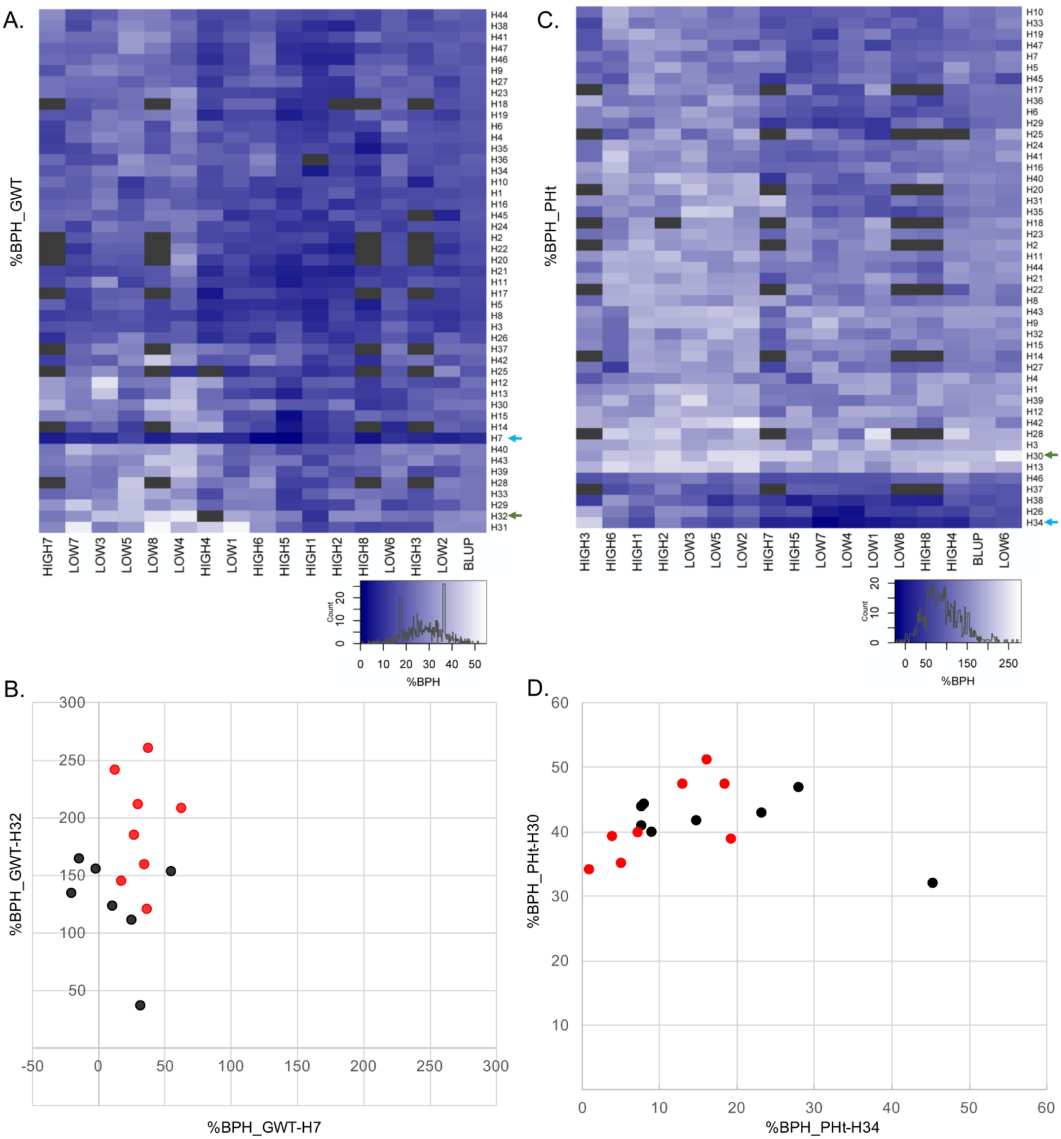
Percent better parent heterosis (%BPH) for grain weight (GWT) and plant height at maturity (PHt) for 47 hybrids across 16 environments. A and C) Heatmap of %BPH for GWT (A) and PHt (C); black shaded cells indicate missing data. The green and blue arrow in each plot indicates the hybrids that have the highest and lowest %BPH across all 16 environments. Environments and hybrids were clustered using hierarchical clustering (trees not shown). B and D) Highest (indicated by green arrows in A and C) vs. lowest (indicated by blue arrows in A and C) %BPH hybrids across all environments for GWT (B) and PHt (D). Red dots are the eight low-density environments and black dots are the eight high-density environments. H7 is PHP02 x DK3IIH6, H30 is LH185 x DK3IIH6, H32 is LH198 x LH185, H34 is LH82 x W606S.

There were differences in the patterns of BPH among environments observed for GWT and PHt (Fig 4A and 4C). GWT generally expressed a greater BPH in low planting density environments, while planting density seemed to have little impact on BPH of PHt. For GWT, the correlation of IBS and BPH at high density was slightly higher (r = -0.58***) than for low density (r = -0.52***) indicating that BPH may be more affected by IBS at high density environments. However, both are highly correlated and IBS is affecting BPH under both conditions. For each of the traits the stability and average BPH was quite variable among hybrids (Fig 4). The hybrid that expressed the lowest and highest BPH values across all the environments were identified for each trait (indicated by arrows in Fig 4A and 4C). The stability of heterosis in these hybrids was evaluated across environments. Interestingly, for PHt the hybrid with the highest BPH exhibited consistently high levels of BPH while the hybrid with the lowest average BPH exhibited quite variable heterosis among environments (Fig 4D). However, this hybrid also had lower hybrid performance and therefore this result may be due to sensitivity to variable neighbor effects. The opposite pattern was observed for the hybrids with highest and lowest average BPH for GWT (Fig 4C). This trend was consistent across the entire set of 47 hybrids (S1 Fig). This may suggest that hybrids with the highest potential for GWT are the most responsive and have higher potential to take advantage of favorable environments.

**Fig 5:**
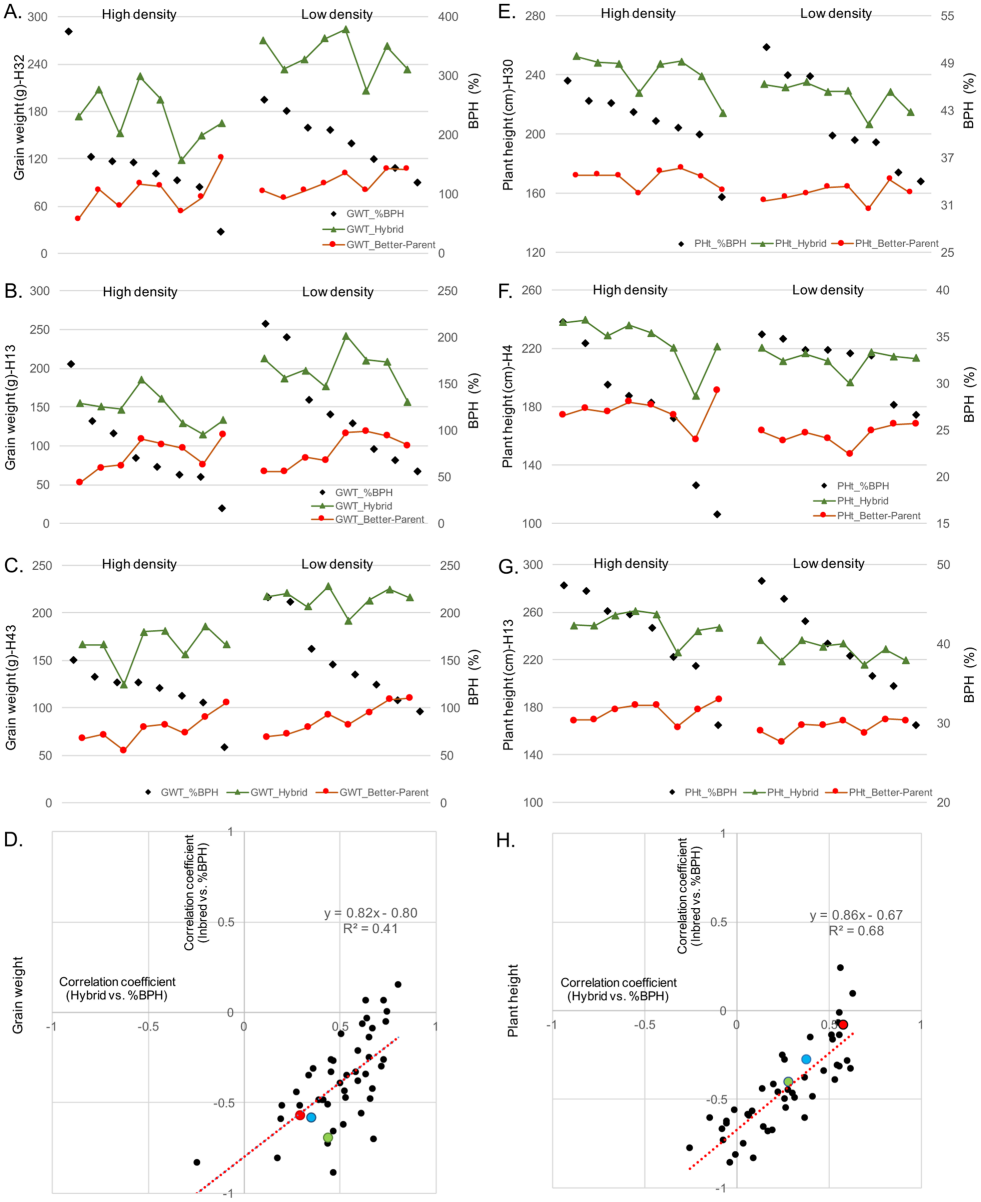
Relationships among percent better parent heterosis (%BPH), hybrid, and better-parent performance. Plots A-D are for grain weight (GWT) and E-H are for plant height at maturity (PHt). A and E) Hybrids with the highest %BPH across 16 environments. B and F) Hybrids with the highest standard deviation of the rank of %BPH among all 47 entries. C and G) Hybrids with the lowest standard deviation of the rank of %BPH among all 47 entries. D and H) Correlation coefficient of hybrid vs. %BPH and better-parent vs. %BPH across 16 environments for each hybrid. Colored dots represent the highest %BPH (red—A and E), highest standard deviation of the rank of %BPH (green—B and F), and lowest standard deviation of the rank of %BPH (blue—C and G). For A-C and E-G dots along the x-axis represent each of the 16 environments.

BPH is a measure of the difference in performance of the hybrid relative to the parents. Environmental influences on BPH could reflect changes in hybrid performance, changes in inbred performance or a combination of both. We investigated the patterns of BPH, hybrid performance and inbred performance for GWT and PHt in a selected set of hybrids (Fig 5). We first assessed the patterns for the hybrids with the highest average BPH for GWT (Fig 5A) and PHt (Fig 5E). We also assessed the patterns for the hybrid with the greatest (Fig 5B and 5F) or least (Fig 5C and 5G) standard deviation for BPH ranks among the environments. These reveal a variety of patterns in the trend of inbred and hybrid performance relative to variation in BPH values among environments. There are examples, such as Fig 5E, in which the reduction in heterosis in some environments is due to reduced hybrid performance with relatively stable inbred performance. In other examples, such as Fig 5C, the changes in heterosis seem to be driven by changes in the inbred performance among the environments.

We proceeded to assess the relative contribution of variation in the inbreds and hybrids to the environmental variation for BPH for all 47 hybrids for GWT and PHt (Fig 5D and 5H). The correlation of better parent performance and BPH (y-axis) was plotted against the correlation of hybrid and BPH (x-axis). As expected, the performance of the better parent tends to be negatively correlated with heterosis while the performance of the hybrid is positively correlated with heterosis. The relative slope of the regression line across different traits in this plot is an indicator of the relative contribution of better parent performance and hybrid performance to heterosis variation. There were differences in the distribution of the correlation values for GWT (Fig 5D) and PHt (Fig 5H). For GWT, 46 of 47 hybrids have a positive correlation between hybrid performance and heterosis (Fig 5D) suggesting that heterosis for GWT is influenced by hybrid performance in all genotypes. In contrast, there are a number of hybrids without significant correlations between hybrid performance and heterosis for PHt (Fig 5H).

We assessed the relative influence of better parent and hybrid variation on BPH for all 25 traits measured in this study (S6 Table). In the majority of cases the hybrid performance is positively correlated with heterosis while the better parent performance is negatively correlated with heterosis. However, the relative strength of the correlations varied among different traits. For traits such as kernel depth, PHt, cob width there was a much stronger correlation between better parent performance and heterosis. Environmental variation for heterosis for other traits such as CWT, KW, and EL are more strongly influenced by the hybrid performance (S6 Table). Interestingly, GWT showed equal strength of correlation for both hybrid performance with heterosis and better parent performance with heterosis. There was, however, a significant negative correlation between “noise” (residual from ANOVA using BPH) and the correlation of better parent performance and BPH (r = -0.77***), which may impact the ability to accurately assess the relative contribution of inbreds and hybrids to observed BPH.

Corn yields have increased continuously since hybrids were first commercially grown in the 1930s. However, the increase in yield of commercial hybrids has not been attributed to an increase in heterosis [46]. Indeed, the percentage of heterosis has not changed substantially, and by some estimates has decreased slightly over time due to the higher percentage rate of gain in yield for inbred lines relative to hybrids [47, 48]. Our data suggest that variation in the performance of both inbred lines and hybrid lines in different environments will influence the amount of heterosis. The relative influence of hybrid variation and inbred variation on heterosis is variable across the traits that were measured in this study. It is worth noting that in some extreme environments inbred lines may be severely affected while hybrids are not, and this outcome will influence measures heterosis [49]. However, in the moderate environments surveyed in this study we find important contributions of both hybrid and inbred performance to heterosis variation.

## Supporting information

S1 Fig Relationship between percent better parent heterosis (%BPH) and variance across environments for the 47 hybrids. Variance was determined as the standard deviation (SD) of performance across the 16 environments. %BPH is the value across all 16 environments.
(TIFF)

S1 Table Inbred and hybrid genotype information. H1-H47 indicate hybrid genotypes that were generate with the parent in the row as the female and the parent in the column as the male.
(XLSX)

S2 Table Meta data on environments used in the study and the traits measured in each environment. (XLSX)

S3 Table Raw phenotypic data for 25 traits measured on 11 inbreds and 47 hybrids at 16 environments. (XLSX)

S4 Table Analysis of variance summary for analysis of inbreds, hybrids, and BPH. Mean squares are reported and * significant at p = 0.05; ** significant at p = 0.01; *** significant at p = 0.001; N.S. not significant.
(XLSX)

S5 Table The corresponding heterotic group of the hybrids in each cluster in Fig 1C. (XLSX)

S6 Table Pearson correlation of %BPH and hybrid performance, inbred performance for 25 traits under high, low and combined density, respectively and broad sense heritability of each trait. Only significant correlations are shown in the table. Red and gray colors correspond to negative and positive values, respectively.
(XLSX)
