# Correction: Genotype-by-environment interactions affecting heterosis in maize

**DOI:** 10.1371/journal.pone.0219528

**Published:** 2019-08-05

**Authors:** Zhi Li, Lisa Coffey, Jacob Garfin, Nathan D. Miller, Michael R. White, Edgar P. Spalding, Natalia de Leon, Shawn M. Kaeppler, Patrick S. Schnable, Nathan M. Springer, Candice N. Hirsch

There is an error in the presentation of the data in S3 Table that caused the phenotypic data for the ear morphology traits to not be matched to the correct plots in the table. All files used for data analyses were correct. Please see the correct S3 Table here.

## Supporting information

S3 Table Raw phenotypic data for 25 traits measured on 11 inbreds and 47 hybrids at 16 environments. (XLSX)

## References

[1] Li Z, Coffey L, Garfin J, Miller ND, White MR, Spalding EP, et al (2018) Genotype-by-environment interactions affecting heterosis in maize. PLoS ONE 13(1): e0191321 10.1371/journal.pone.0191321 29342221 PMC5771596

