# Supplementary figures and images for: Genotype-by-environment interactions affecting heterosis in maize
Source: PLoS One. 2018 Jan 17;13(1):e0191321. doi: 10.1371/journal.pone.0191321 (PMC5771596; doi:10.1371/journal.pone.0191321)

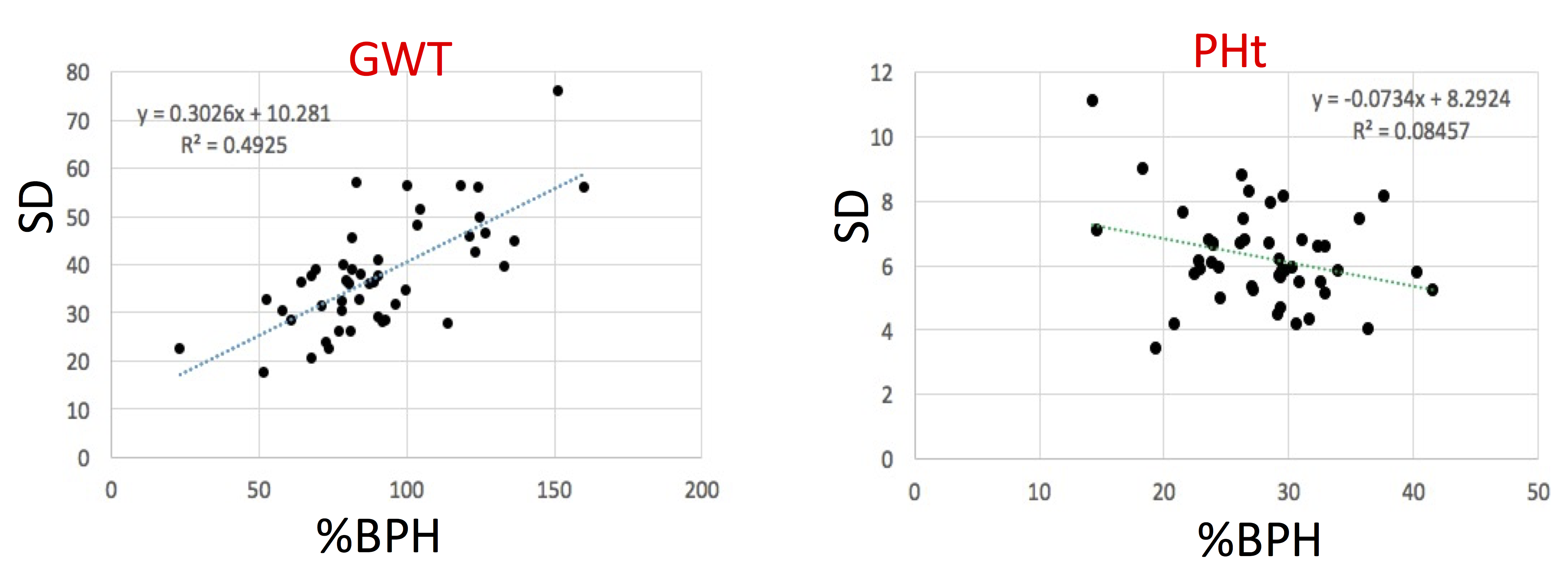

Supplement: S1 Fig — Variance was determined as the standard deviation (SD) of performance across the 16 environments. %BPH is the value across all 16 environments. (TIFF) [file pone.0191321.s001.tiff]
